# *Coffea arabica* pulp aqueous extract exhibits the anti-colitogenic effect in mice: preventive efficacy and possible mechanisms of action

**DOI:** 10.1186/s40659-026-00696-9

**Published:** 2026-05-20

**Authors:** Apiwan Arinno, Pichayapa Sukmak, Supisara Treveeravoot, Purit Kulworasreth, Pitsinee Supapol, Withsakorn Sangsuwan, Natnicha Teansuk, Wanapas Wachiradejkul, Jakkapong Inchai, Thaniya Sricharunrat, Kanthida Jangyubol, Patthadon Sanghong, Chutima S. Vaddhanaphuti, Chanat Aonbangkhen, Pawin Pongkorpsakol

**Affiliations:** 1 Center of Excellence in Natural Products Chemistry (CENP), Department of Chemistry, Faculty of Science, Chulalongkorn University, Bangkok, Thailand; 2 Center of Excellence on Petrochemical and Materials Technology, Chulalongkorn University, Pathumwan, Bangkok, Thailand; 3 Laboratory of Epithelial Tight Junction Pathophysiology, Bangkok, Thailand; 4 Department of Science, Technology and Innovation, Faculty of Science, Chulabhorn Royal Academy, Bangkok, Thailand; 5 Princess Srisavangavadhana Faculty of Medicine, Chulabhorn Royal Academy, Bangkok, Thailand; 6 International Collaborative Medical Research Laboratory, Princess Srisavangavadhana Faculty of Medicine,, Chulabhorn Royal Academy, Bangkok, Thailand; 7 Innovative Research Unit of Epithelial Transport and Regulation (iETR), Department of Physiology, Faculty of Medicine, Chiang Mai University, Chiang Mai, Thailand; 8 Chulabhorn Hospital, Chulabhorn Royal Academy, Bangkok, Thailand; 9 Cytogenetics Research Laboratory, Bureau for Research and Innovation Management, Chulabhorn Royal Academy, Bangkok, Thailand; 10 CRA Excellent Centre for Drug Discovery, Bureau for Research and Innovation Management, Chulabhorn Royal Academy, Bangkok, Thailand

**Keywords:** *Coffea arabica* pulp aqueous extract (CPE), Tight junction, Intestinal barrier function, Sirtuin-1 (SIRT-1), Colitis

## Abstract

Inflammatory bowel disease (IBD) is a chronic inflammatory disease caused mainly by immune overactivation and intestinal mucosal barrier disruption. Coffea arabica pulp aqueous extract (CPE) contains a number of bioactive phenolic compounds that exhibit antioxidant and anti-inflammatory effects with previously unexplored application in experimental colitis. The aim of this study is to determine whether CPE produces anti-colitogenic effect and its possible mechanism of action. We found that CPE attenuated all IBD-related phenotypes and increased survival rates in dextran sulfate sodium (DSS)-induced colitis mice. In addition, CPE significantly suppressed mRNA and protein expression of myosin light-chain kinase (MLCK). Furthermore, CPE also inhibited MLCK recruitment to apical junction of colonic tissues of colitis mice. Although CPE had no effect on mRNA expression of tight junction genes, it reversed inflammation-mediated downregulation of ZO-1, occludin, and claudin-4 proteins in colitis mice. Importantly, CPE was able to recover ZO-1 and occludin localization to apical junction and suppressed tight junction-dependent leak pathway permeability in colitis mice. Indeed, CPE was also capable of stimulating sirtuin-1 (SIRT-1) in colonic tissues obtained from DSS-induced colitis mice, which is known to suppress inflammation and enhance intestinal barrier function. Therefore, SIRT-1 has been shown to be associated with CPE treatment in IBD model.

**Graphical abstract:**

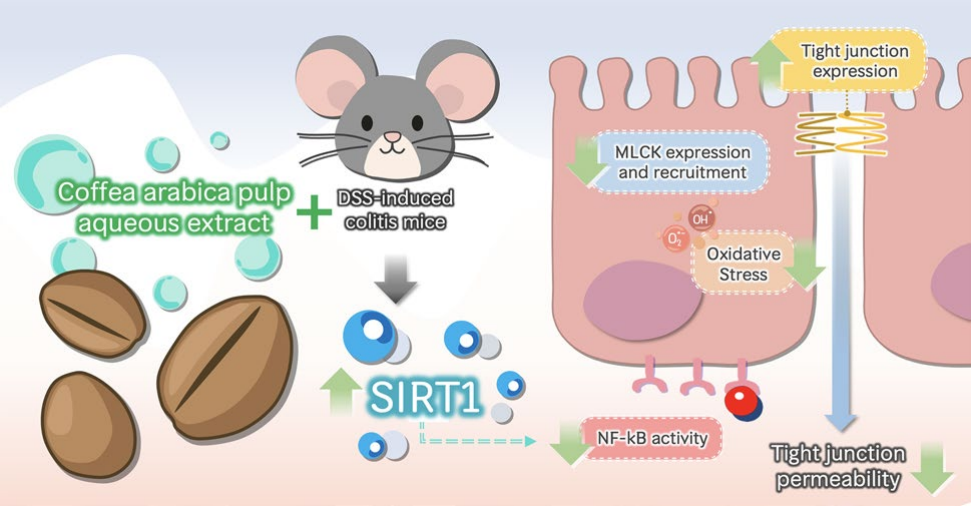

**Supplementary Information:**

The online version contains supplementary material available at 10.1186/s40659-026-00696-9.

## Introduction

Inflammatory bowel disease (IBD) is considered as a group of autoimmune-mediated, indefinite colitis [8, 20]. It can be divided into ulcerative colitis (UC) and Crohn’s disease (CD), which are characterized by mucosal damages of colon to rectum and throughout the gastrointestinal tract [21, 23, 47], respectively. Several factors including intestinal tight junction defects contribute to IBD pathophysiology [9, 10, 13, 19, 36, 38, 42, 50, 68]. Oral 5-aminosalicylate (5-ASA) administration, the first-line treatment, is not effective in severe cases [41]. Corticosteroid can treat IBD with risks of infection and cardiovascular adverse effects [18].

Expression of several cytokines have been found in colonic biopsies of IBD patients [1, 35, 60, 62]. Intestinal permeability and IBD-related cytokine expression were found in healthy first-degree relatives of Crohn’s disease patients [5, 30, 57]. In response to cytokines, myosin light-chain kinase (MLCK) expression and recruitment to perijunctional actomyosin ring promotes tight junction disruption-dependent leak pathway permeability, with having paracellular hydrodynamic radius of leaky area of approximately 14 angstrom [16, 60–62, 71]. Of note, 4-kDa FITC-dextran, but not 70-kDa FITC-dextran, can pass through leak pathway permeability [7]. In case of tissue damage, paracellular permeability of 70-kDa FITC-dextran is increased via apoptosis-associated unrestricted pathway permeability [7]. Recovery of tight junction disruption attenuated colitis in mouse models [16, 52, 71]. Therefore, tight junction has been considered as an effective drug target for IBD [32]. No FDA-approved drug to rescue intestinal tight junction disruption was reported. Recently, we found that activation of sirtuin-1 (SIRT-1) enhanced tight junction assembly in colonocytes [53]. Hence, SIRT-1 has been proposed as a recently emerging drug target for tight junction recovery.

*Coffea arabica L.* is one of the most popular beverages. Although coffee pulp (CP) is an agricultural waste product of industrial coffee processing, its aqueous extract contains a number of phenolic compounds including caffeine, anthocyanins, catechin, epicatechin, and chlorogenic acid (CGA) [4, 12, 39, 65]. Coffee pulp aqueous extract (CPE) exhibited antioxidant and antimicrobial activities [22, 65]. Hypolipidemic effects of CGA was shown in hypercholesterolemic rats [39, 49]. Epicatechin predominantly reduced blood glucose in streptozotocin-induced diabetic rats [4, 45]. Interplay between intracellular metabolic pathways and tight junction assembly has been accumulatively documented [32, 43, 53, 66], it is possible that CPE may confer protective effects in experimental colitis but has never been explored. Here, we show that CPE attenuated severity of experimental colitis mice.

## Materials and methods

### Experimental colitis mice and treatment with CPE

All experiments in this study used C57BL/6 male mice. Indeed, C57BL/6 mice were obtained from the Nomura Siam International Co.,Ltd. and maintained at the Central Animal Facility (MUSC-CAF) Faculty of Science, Mahidol University in accordance with the regulations of the Institutional Animal Care and Use Committee (IACUC) (Protocol No. MUSC66-055-685). For induction of experimental colitis, dextran sulfate sodium (DSS; 5% in drinking water) (molecular weight of batch of DSS is approximately 40 kDa, Cat. #J63606.22, Thermo Fisher Scientific Inc., Waltham, MA, USA) was used as a colonic tissue-sensitive irritant agent to promote acute experimental colitis for 8 days (for disease activity, molecular and histopathological analyses) or 14 days (for survival study) as previously described [69]. To demonstrate the pharmacological property of CPE, the DSS-induced colitis mice were intra-gastrically administered with CPE at various doses (10, 50, and 100 mg/kg) from the first day to the end of the experiment. The protective efficacy of CPE was evaluated by clinical symptoms and survival rate of mice. Moreover, all colonic tissues of mice were further analyzed using molecular techniques including quantitative real-time PCR and western blot analysis etc.

### Preparation of CPE

Coffee pulp extract (CPE) used in this study was derived from dried coffee pulp, which was kindly provided by Hillkoff (Chiang Mai, Thailand). In short, dried coffee pulp was infused by hot water for 10 min. The liquid extract was filtered thrice, concentrated and lyophilized using a rotary evaporator and a lyophilizer, respectively.

### Clinical assessment of DSS-induced colitis mice treated with CPE

Severity and progression of colitis in mice induced by DSS treatment were evaluated daily using the disease activity index (DAI) that was scored from 0 to 2 each for posture, fur texture, motor activity, and diarrhea (0–8) as described previously [16, 46, 52]. Colonic tissues damage was investigated based on colon shortening and basic histopathological analysis (depending on epithelial damage, architectural change, crypt abscess, mucin depletion, and submucosal edema) of the colon tissues. In addition, colonic inflammation and immune cell infiltration was investigated using Nancy index as previously described [59]. Of note, histopathological analysis and evaluation of Nancy index were done by an anatomical pathologist who is blinded to the experimental conditions.

### Myeloperoxidase (MPO) activity assay

Neutrophils that highly expresses MPO can generally infiltrate into inflamed colonic mucosa in IBD patients. MPO activity of colonic tissue samples that represents local neutrophil infiltration can be quantitively measured by MPO activity assay (Cat. #ab105136, Abcam, Cambridge, MA, USA.). In brief, MPO can generate hypochlorous acid (HClO) that can produce taurine chloramine, which oxidizes a yellow chromogenic TNB probe to reduce its chromatic signals. The optical density (O.D.) for absorbance measurement of TNB was able to be measured at wavelength of 412 nm. Indeed, O.D. value of TNB is inversely proportional to the amount and activity of MPO enzyme.

### Measurement of transcript expression of cytokine-associated IBD and tight junction

Quantitative reverse transcription PCR (RT-qPCR) was performed to evaluate transcript expression of cytokines that are known to contribute to pathogenesis of IBD including TNF, IFN-γ, IL-1β, IL-6, and IL-8 as well as transcripts encoding tight junctions. Total RNA was extracted from mouse colon tissues using RNeasy Mini Kit (Cat #74106) (Qiagen, Hilden, Germany). Concentration of RNA samples were measured using NanoDrop (NanoDrop Technologies Inc.,Wilmington, DE, USA). Synthesis of cDNA from RNA templates was performed using an iScript Reverse Transcription Supermix (Cat. #1708841, Bio-Rad Laboratories, Inc., Hercules, CA, USA). Amplifications were performed using the CFX Opus 96 Real-Time PCR System (Bio-Rad Laboratories, Inc., Hercules, CA, USA) with PowerTrack™ SYBR™ Green Master Mix (Cat. #A46109, Applied Biosystem, Foster City, CA, USA). The primer sets used in this study were shown in supplementary Table 1.

### Western blotting analysis

Colonic tissues of normal and DSS-treated mice with or without CPE (100 mg/kg) were collected to be further homogenized in lysis buffer using TissueLyser LT system (Qiagen, Hilden, Germany). Furthermore, expression of MLCK and tight junction proteins including occludin, zonula occludens-1 (ZO-1), claudin-1, and claudin-4 were detected using western blot analysis. Briefly, protein lysates were then loaded and separated using sodium dodecyl sulfate polyacrylamide gel electrophoresis (SDS-PAGE) and further transferred to a nitrocellulose membrane by the Trans-Blot Turbo transfer system (Bio-Rad Laboratories, Hercules, CA, USA). To eliminate the non-specific binding band artifact of the antibodies, membrane containing proteins was blocked for few minutes with BlockPRO™ 1 Min Protein-Free Blocking Buffer (Cat. #BM10-100, Visual Protein, Neihu Dist., Taipei, Taiwan). Moreover, membrane containing protein samples were incubated with specific primary antibodies against target proteins overnight (4 °C) and secondary antibodies for an hour (room temperature). For detecting signals, luminol/enhancer solution and peroxide (Cat. #1705060)(Bio-Rad Laboratories, Hercules, CA, USA) was used. Band densitometry of protein expression of target proteins were analyzed using Image Lab Software (Bio-Rad Laboratories, Hercules, CA, USA). Lists of primary and secondary antibodies used for western blot analysis were shown in supplementary Table 2.

### Immunofluorescence staining

To visualize localization of tight junctions and MLCK at the apical surface of colonic tissues and nuclear factor kappa B (NF-κB) at cytoplasmic-to-nuclear space, immunofluorescence staining was performed. Briefly, colonic tissues of normal and DSS-treated mice with or without CPE (100 mg/kg) were fixed with 4%paraformaldehyde in PBS and further embedded in paraffin blocks. The colonic tissues in paraffin blocks were then cut into 5 μm-thick sections, de-paraffinized in xylenes, and further dehydrated with through graded alcohols. Tissue sections were stained with primary antibodies against target proteins of interests and fluorescently-conjugated secondary antibodies. Fluorescent signals were visualized by FV3000 confocal microscope (Olympus, Japan). Lists of primary and secondary antibodies used for immunofluorescence staining were shown in supplementary Table 2.

### SIRT-1 activity assay

Enzymatic activity of NAD^+^-dependent SIRT1 of normal and DSS-treated mice with or without CPE (100 mg/kg) was measured using a SIRT1 Activity Assay Kit (ab156065, Abcam, MA, USA) according to kit instruction and adapted from previous study [53]. Protein lysates of normal and colitis mice were mixed with SIRT1 Assay Buffer, Fluoro-Substrate Peptide, and NAD. The fluorescent signals of reaction were detected using a microplate reader at excitation/emission wavelength of 350 nm/450 nm at 2-minute intervals for 20 min.

### In vivo paracellular permeability assay

To distinguish between tight junction-dependent leak pathway permeability and tight junction-independent, tissue damage-associated barrier loss in experimental colitis mice, in vivo multiplex permeability assay was performed and slightly adapted according to the stepwise protocol previously described [7]. In short, permeability probe cocktail containing 4-kDa FITC-dextran (80 mg/ml) (Cat. #FD4, Millipore-Sigma) and 70 kDa rhodamine-dextran (40 mg/ml) (Cat. #R9379, Millipore-Sigma) was prepared in ultrapure water. To empty the remaining food in the GI tract, fasting was performed three hours before starting the experiment. Permeability probe cocktail was gently administrated to normal, DSS-induced colitis mice with or without CPE (100 mg/kg) by intragastric gavage. At 3 h after gavage, blood sample collection was performed by cardiac puncture. Fluorescent intensity of mouse serum samples from each experimental group were evaluated using Biotek Synergy HT. Fluorescein and rhodamine B fluorescence were read at Excitation/Emission wavelength of 495 nm/525 nm and 555 nm/585 nm, respectively.

### Statistical analysis

All presented data are expressed as means ± S.E.M. Statistical analysis for multiple comparisons in this study was determined by the analysis of variance (one- or two-way ANOVA) followed by the Bonferroni analysis using Prism 5.0, where appropriate. For body weight and DAI, data were analyzed using repeated-measures analysis followed by Tukey’s multiple comparison test. Data points after death were treated as missing and were not imputed and the effective sample number may therefore vary at later time points in groups with mortality. For survival studies, Log-rank (Mantel-Cox) test was performed. If *P*-value was < 0.05 is considerably statistically significant. Statistical analyses for all experiments of this study were tested by GraphPad Prism software.

## Results

### Protective effect of CPE against DSS-induced colitis in mice

**Fig. 1:**
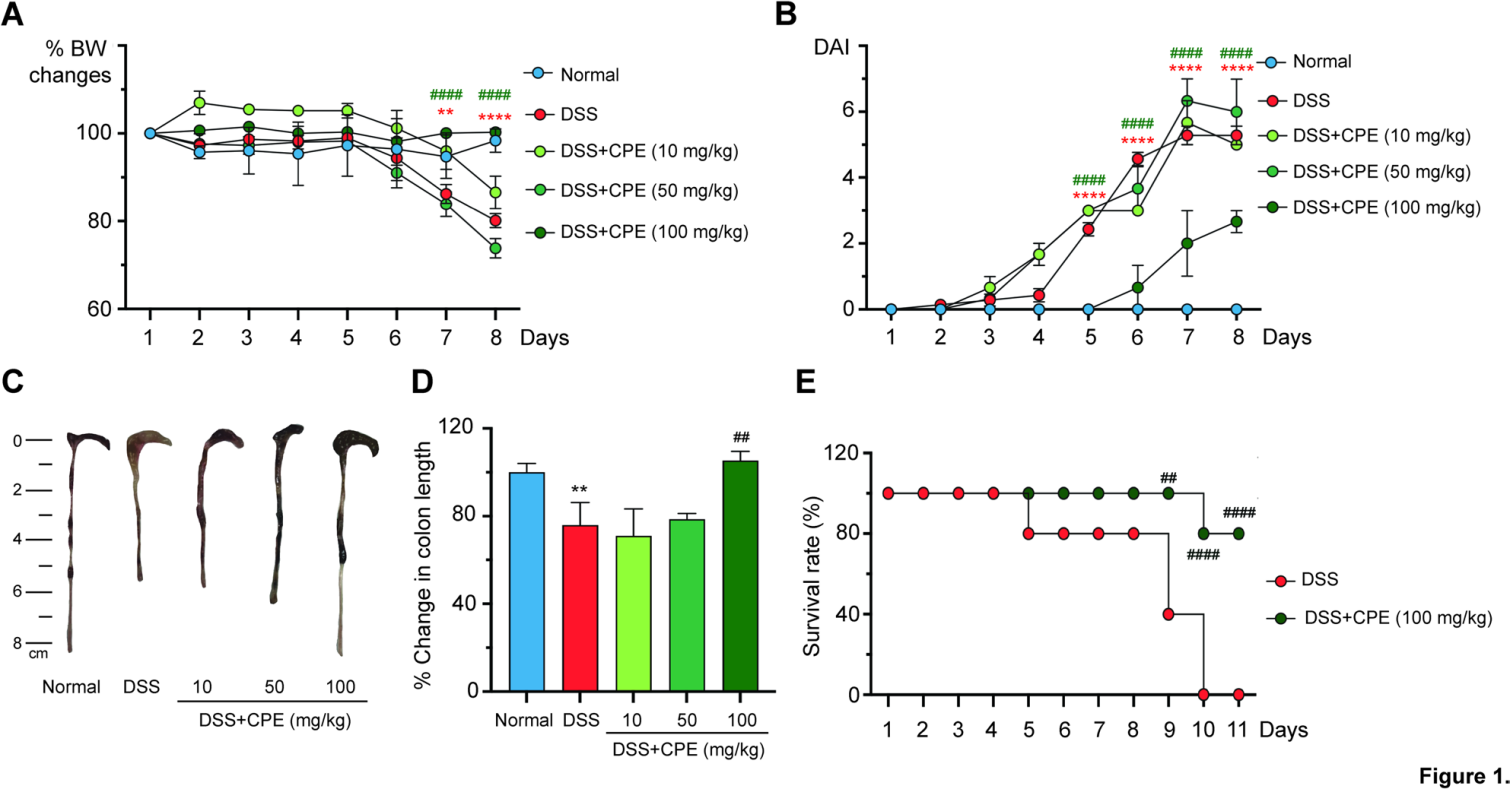
Treatment with CPE attenuated all IBD-related clinical symptoms of DSS-induced colitis. Mice were orally administered with CPE at 10, 50, and 100 mg/kg/day in parallel with feeding 5% dextran sulfate sodium (DSS) in drinking water. **A** Percent body weight change of DSS-induced colitis mice over time compared to normal and CPE-treated groups. **B** Disease activity index (DAI) of DSS-induced colitis mice with CPE administration compared to vehicle-treated group. **C** Representative photographs of the effect of CPE treatment on the colon length in DSS-induced colitis mice. **D** Summary of changes in colon length at day 8. **E** Effect of CPE treatment on survival rate of mice at 14 days after induction of DSS-induced colitis. Data were expressed as mean ± SEM (*n* = 6). **p < 0.01; ****p < 0.0001 compared with normal mice. ##*p* < 0.01; ####*p* < 0.0001 compared with DSS-treated mice

Preventive effect of CPE in the treatment of IBD was initially determined in the DSS-induced experimental colitis mouse model. In this experiment, mice were given 5% DSS in drinking water with or without daily oral administrations of various concentrations of CPE for 8 days. As depicted in Fig. 1A and B, at the end of the experiment, CPE (100 mg/kg/day) was capable of significantly diminishing the clinical hallmarks of colitis including body weight loss and disease activity index (DAI) compared to vehicle-treated, DSS-induced colitis mice and normal. Moreover, the effects of CPE on clinical severities of DSS-induced experimental colitis mice were further demonstrated according to colon length and survival rate. Surprisingly, CPE dose-dependently reversed DSS-induced colon length shortening in mice (Fig. 1C and D). Of particular importance, CPE (100 mg/kg/day) significantly enhanced survival rate of DSS-induced colitis mice (Fig. 1E).

### Pathohistological and molecular immune activity analyses of colonic tissues from colitis mice treated with CPE

**Fig. 2:**
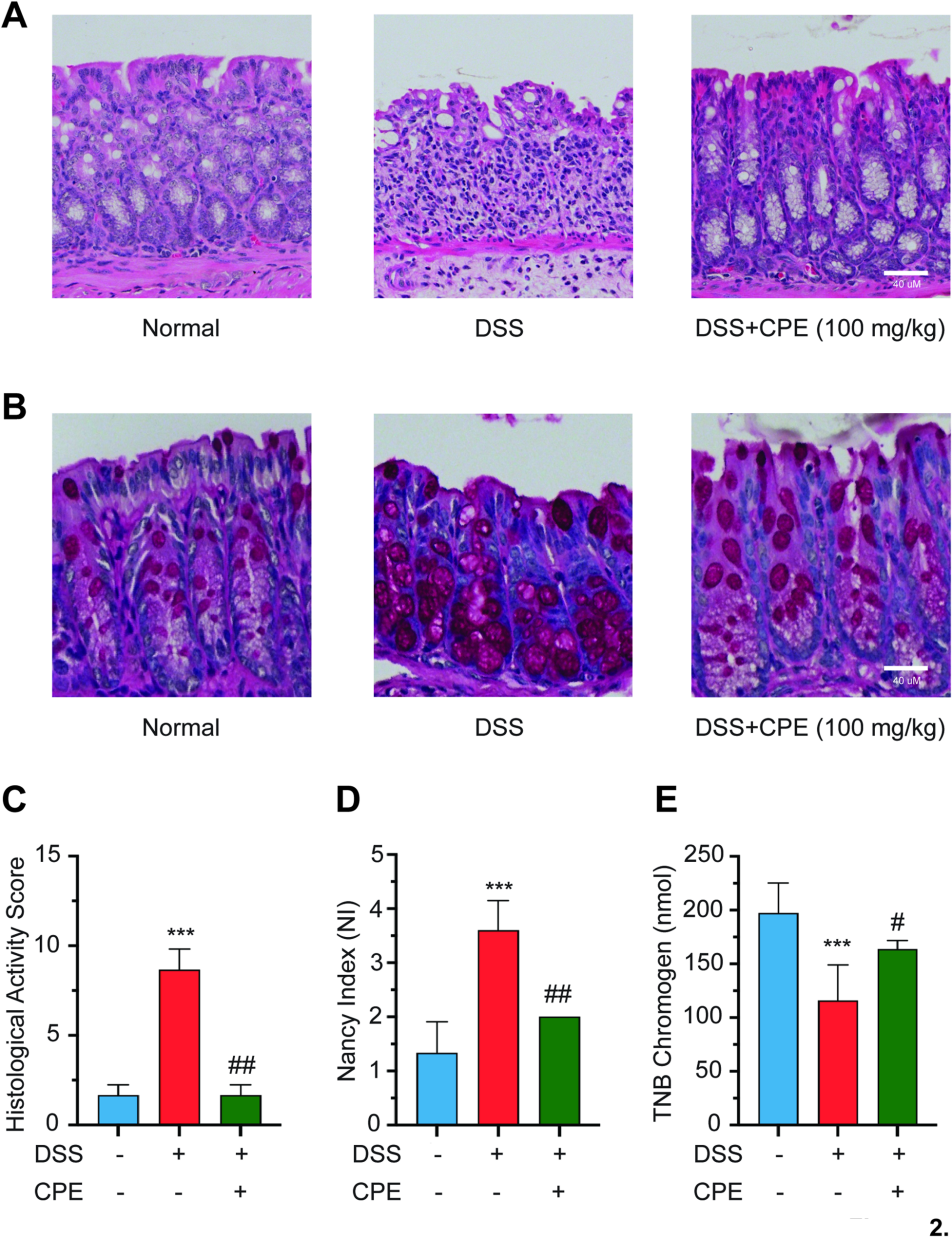
Histopathological analyses of colonic tissues obtained from colitis mice. **A** Representative H&E staining images of colon sections from DSS-induced colitis mice treated with or without CPE as indicated at day 8 were shown (20×). **B** Representative PAS-positive staining of colon sections from DSS-induced colitis mice treated with or without CPE. **C** Summary of the histopathological scores based on morphological changes of the colonic tissue architecture. **D** Summary of the histopathological scores according to Nancy index that observed immune cell infiltration. **E** Levels of TNB chromogen of proteins obtained from colonic tissues of DSS-induced colitis mice that represented the activity of neutrophil infiltration. Data were expressed as mean ± SEM (*n* = 6). ***p < 0.001 compared with normal mice. #*p* < 0.05; ##*p* < 0.01 compared with DSS-treated mice

At 8 days post-administration of DSS and CPE, colon tissues from normal, DSS-fed with or without CPE-treated mice were fixed with 4% paraformaldehyde in PBS and collected in paraffin blocks, tissue blocks were sectioned to be further stained by hematoxylin and eosin (H&E) reagents or periodic-acid Schiff (PAS) reagent. Based on H&E staining results, we found that DSS induced signs of colitis in colonic epithelial tissues including colonic epithelial architectural changes, submucosal edema, and immune cell infiltration (Fig. 2A). Interestingly, CPE attenuated DSS-induced histopathological damages of colonic tissues and immune infiltration (Fig. 2A). In addition, we also found that, in DSS-induced colitis mice, numbers of PAS-positive goblet cells per crypt were predominantly increased, but intensity of PAS staining within goblet cells that indicated amounts of mucin was substantially reduced when compared to normal (Fig. 2B). Indeed, CPE suppressed mucin depletion and reduced PAS-positive goblet cells in DSS-induced colitis mice (Fig. 2B). Based on our H&E and PAS staining, all sectioned colonic tissue slides were further scored by an anatomical pathologist using basic histopathological score and Nancy index. It is noticed that CPE significantly reduced histopathological score and Nancy index in DSS-induced colitis mice (Fig. 2C, D). Neutrophil infiltration to colonic submucosal region has been considered as one of the histopathological hallmarks of IBD patients [11]. In fact, neutrophil highly expresses MPO enzyme that can catalytically reduce TNB chromogen in colonic tissues [48, 69]. It was found that TNB was significantly decreased in protein lysates collected from colonic tissues of DSS-induced mice but was reversed by the treatment of CPE (100 mg/kg/day) (Fig. 2E). These results indicated that CPE treatment was able to rescue histopathological tissues and inflammation in DSS-induced colitis mice.

### Effects of CPE on the function of the inflammatory master regulator and expression of its downstream cytokines in colitis mice

**Fig. 3:**
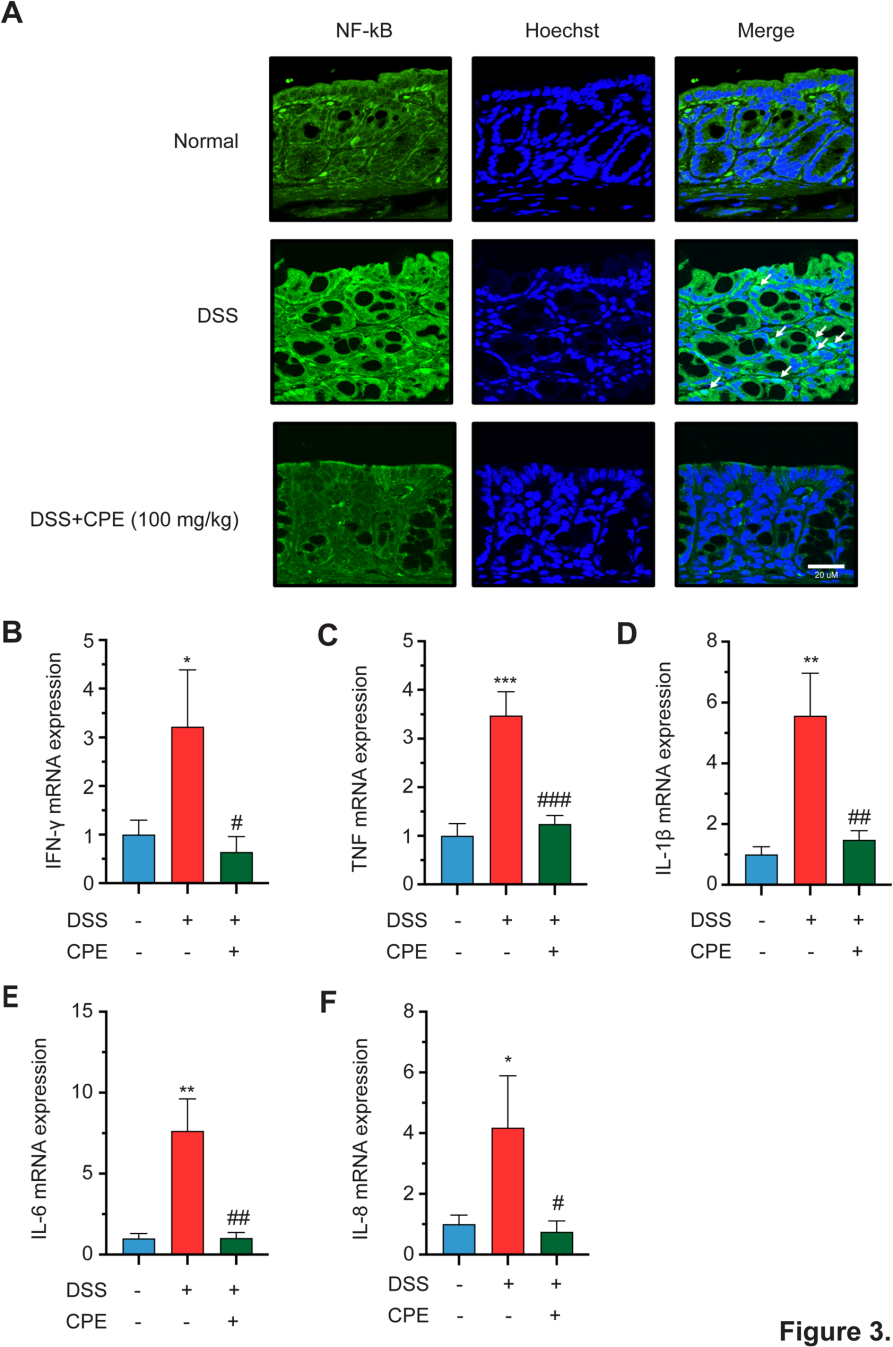
Effects of CPE on NF-κB signaling and its downstream inflammatory cytokine expression in colitis mice. **A** Effect of CPE on NF-κB nuclear translocation, and expressions of **B** IFN-γ, **C** TNF, **D** IL-1β, **E** IL-6, and **F** IL-8 transcripts. Data were expressed as mean ± SEM (*n* = 6). *p < 0.05; **p < 0.01; ***p < 0.001 compared with normal mice. #*p* < 0.05; ##*p* < 0.01; ###*p* < 0.001 compared with DSS-treated mice

In accordance with IBD pathogenesis, NF-κB nuclear translocation has been considered as an early morbific phase resulting in mucosal immune activation and destruction of tissue barriers [3]. To investigate whether CPE suppresses NF-κB nuclear translocation in colitis mice, immunofluorescence staining of NF-κB p65 was performed. As expected, DSS promoted NF-κB p65 accumulation in the nucleus rather than in cytoplasmic space in colonic mucosal tissues of normal mice (Fig. 3A). It was found that CPE predominantly suppressed cytoplasm-to-nucleus translocation of NF-κB p65 (Fig. 3A). It is well accepted that NF-κB is a master regulator of several IBD pathogenesis-related cytokines including TNF, IFN-γ, IL-1β, IL-6, and IL-8 [29]. In order to evaluate the function of NF-κB, expression of its transcripts was measured by quantitative real-time PCR. Here, we found that CPE (100 mg/kg/day) treatment significantly suppressed transcript expression of TNF, IFN-γ, IL-1β, IL-6, and IL-8 (Fig. 3B). These data suggested that CPE suppressed intestinal inflammation, at least in part, by inhibiting NF-κB nuclear translocation-mediated cytokine transcription.

### Effects of CPE on MLCK expression and recruitment to apical junction of colonic mucosa in colitis mice

**Fig. 4:**
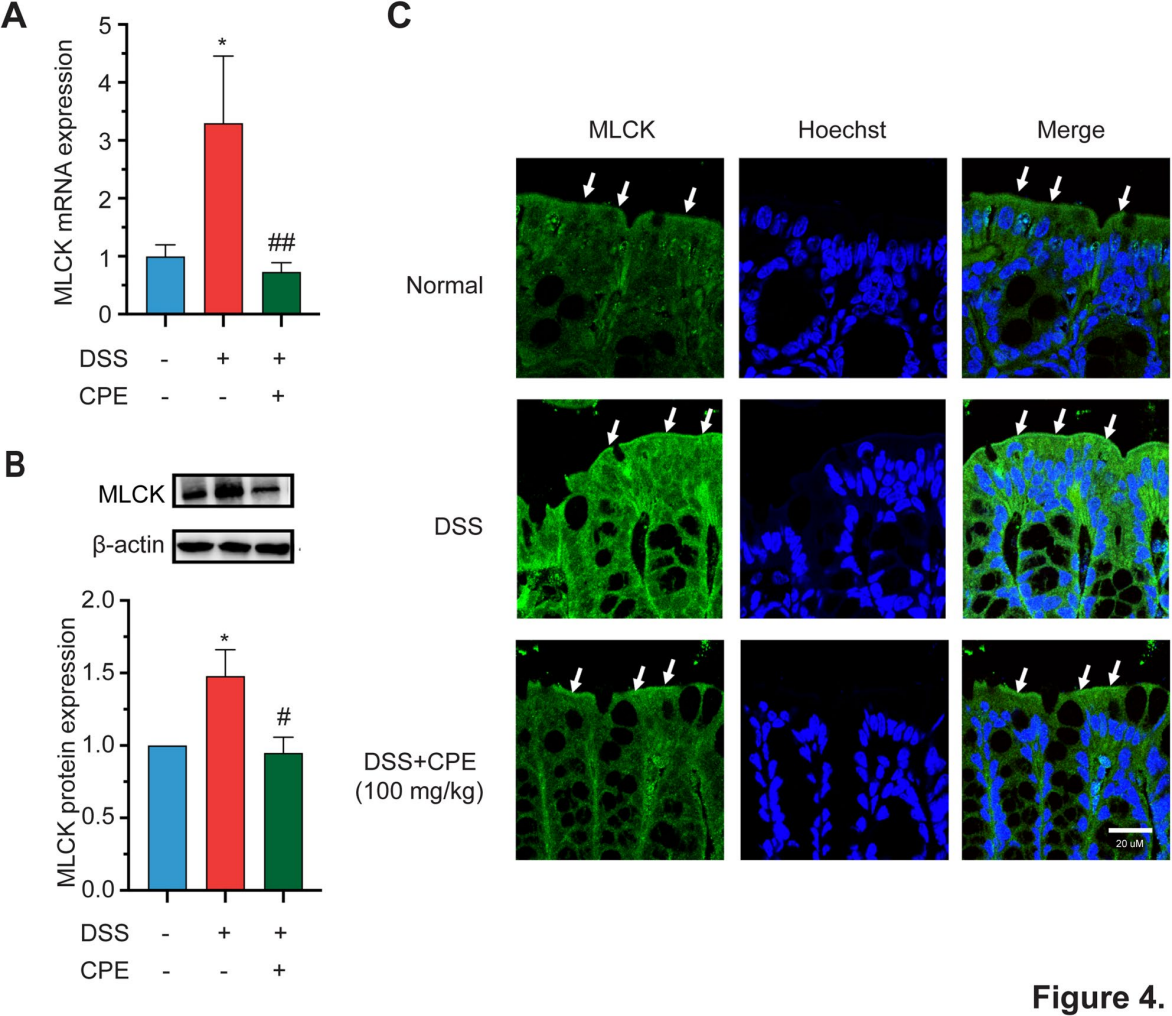
Effects of CPE on MLCK signaling in colonic tissues of DSS-induced colitis mice. Effects of CPE treatment on expressions of **A** MLCK transcript and **B** protein expression as well as **C** MLCK recruitment to apical junction. Data were expressed as mean ± SEM (*n* = 6). *p < 0.05 compared with normal mice. #*p* < 0.05; ##*p* < 0.01 compared with DSS-treated mice

MLCK expression and recruitment to apical junction promotes phosphorylated MLC-mediated contraction of actin cytoskeleton, resulting in intestinal tight junction disruption [2, 16, 71]. We further investigated the involvement of CPE treatment on MLCK function. Therefore, RT-qPCR, western blot analysis, and immunofluorescence staining were performed to provide critical analyses of colonic tissues from DSS-induced colitis mice fed with or without CPE. Indeed, we found that in DSS-induced colitis mice, MLCK transcript and protein were significantly upregulated when compared to normal mice and MLCK was predominantly accumulated at the apical surfaces of colonic tissues of colitis mice as well (Fig. 4A–C). Surprisingly, CPE significantly diminished the levels of transcripts and protein expressions of MLCK in colitis mice (Fig. 4A, B). Expectedly, it was also found that CPE significantly suppressed MLCK recruitment to apical junction of colonic tissues of colitis mice (Fig. 4C). These results suggested that CPE treatment was associated with reduced MLCK expression and preserved tight junction proteins in DSS-induced colitis.

### Effects of CPE on transcription and protein expression of intestinal junctions in colonic tissues of DSS-induced colitis mice

**Fig. 5:**
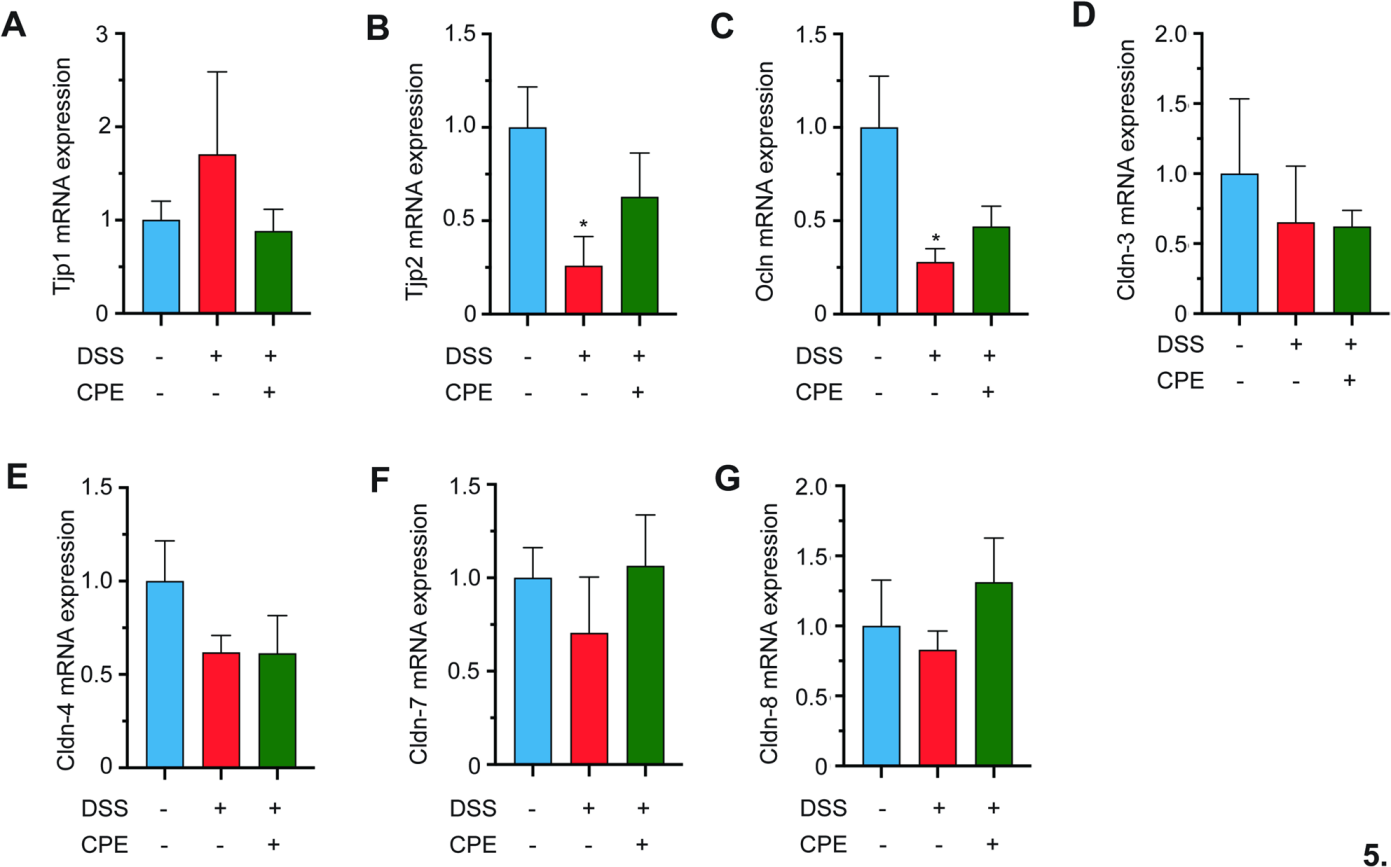
Effects of CPE on tight junction-related transcript expression in colitis mice. The RT-qPCR revealed the mRNA expression levels of **A** *Tjp1*, **B** *Tjp2*, **C** *Ocln*, **D** *Cldn-3*, **E** *Cldn-4*, **F** *Cldn-7*, and **G** *Cldn-8*. Data were expressed as mean ± SEM (*n* = 6). *p < 0.05 compared with normal mice

**Fig. 6:**
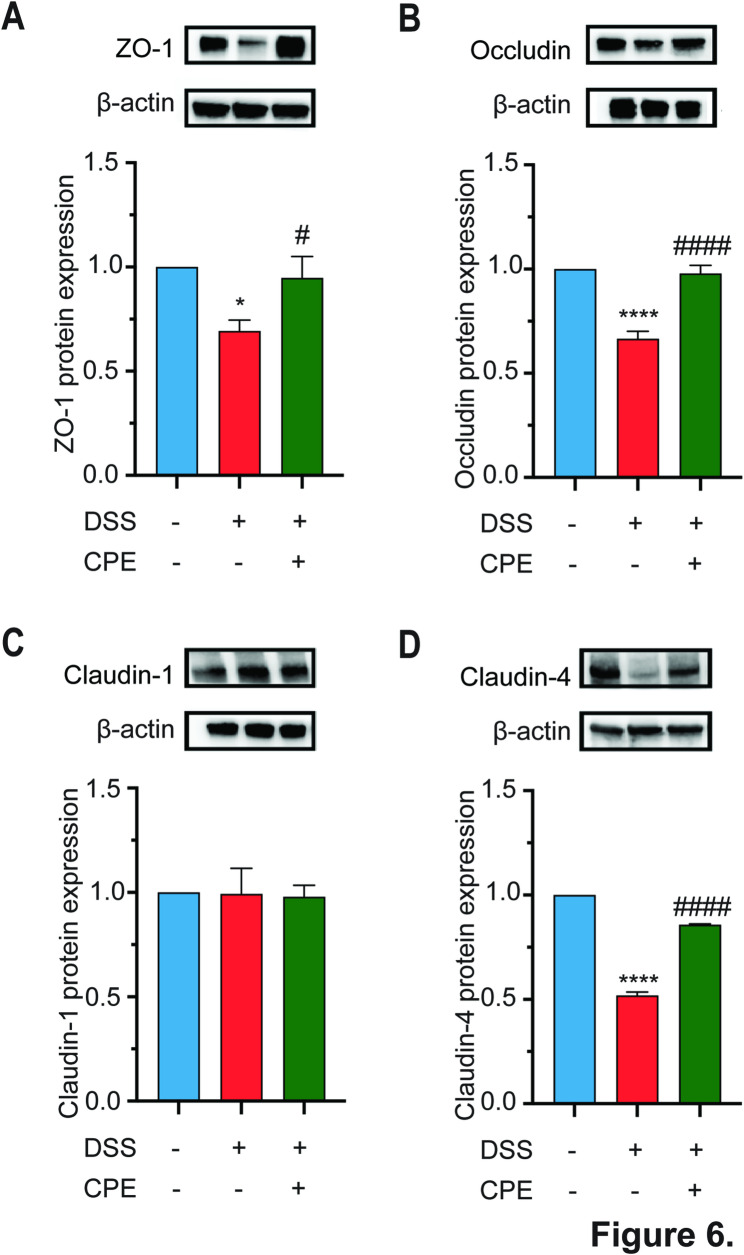
Effects of CPE on tight junction-related protein expression in colitis mice. Western blot analyses indicated the protein expression levels of **A** ZO-1, **B** occludin, **C** claudin-1, **D** claudin-4. *p < 0.05; ****p < 0.0001 compared with normal mice. #*p* < 0.05; ####*p* < 0.0001 compared with DSS-treated mice

To further evaluate the expression levels of tight junction-related transcripts and proteins in colonic tissues from DSS-induced colitis mice treated with or without CPE, quantitative real-time PCR and western blot analyses were performed. Here, we found that mRNA expression of *Tjp1*, *Cldn3*, *Cldn4*, *Cldn7*, *Cldn8* were not changed in DSS-induced colitis mice (Fig. 5A, D, E, F, G). On the other hand, *Tjp2* and *Ocln* transcripts were significantly decreased in DSS-induced colitis mice (Fig. 5B, C). CPE treatment did not, however, reverse intestinal inflammation-suppressed gene expression of *Tjp2* and *Ocln* transcripts in colonic tissues of DSS-induced colitis mice (Fig. 5B, C). In addition, protein expression levels of ZO-1, occludin, and claudin-4, but not claudin-1, were significantly diminished in DSS-induced colitis mice (Fig. 6A–D). Interestingly, CPE treatment significantly increased protein expression of ZO-1, occludin, and claudin-4 when compared to vehicle-treated colitis mice (Fig. 6A, B, D).

### Recovery of intestinal tight junction localization in colitis mice by the treatment of CPE

**Fig. 7:**
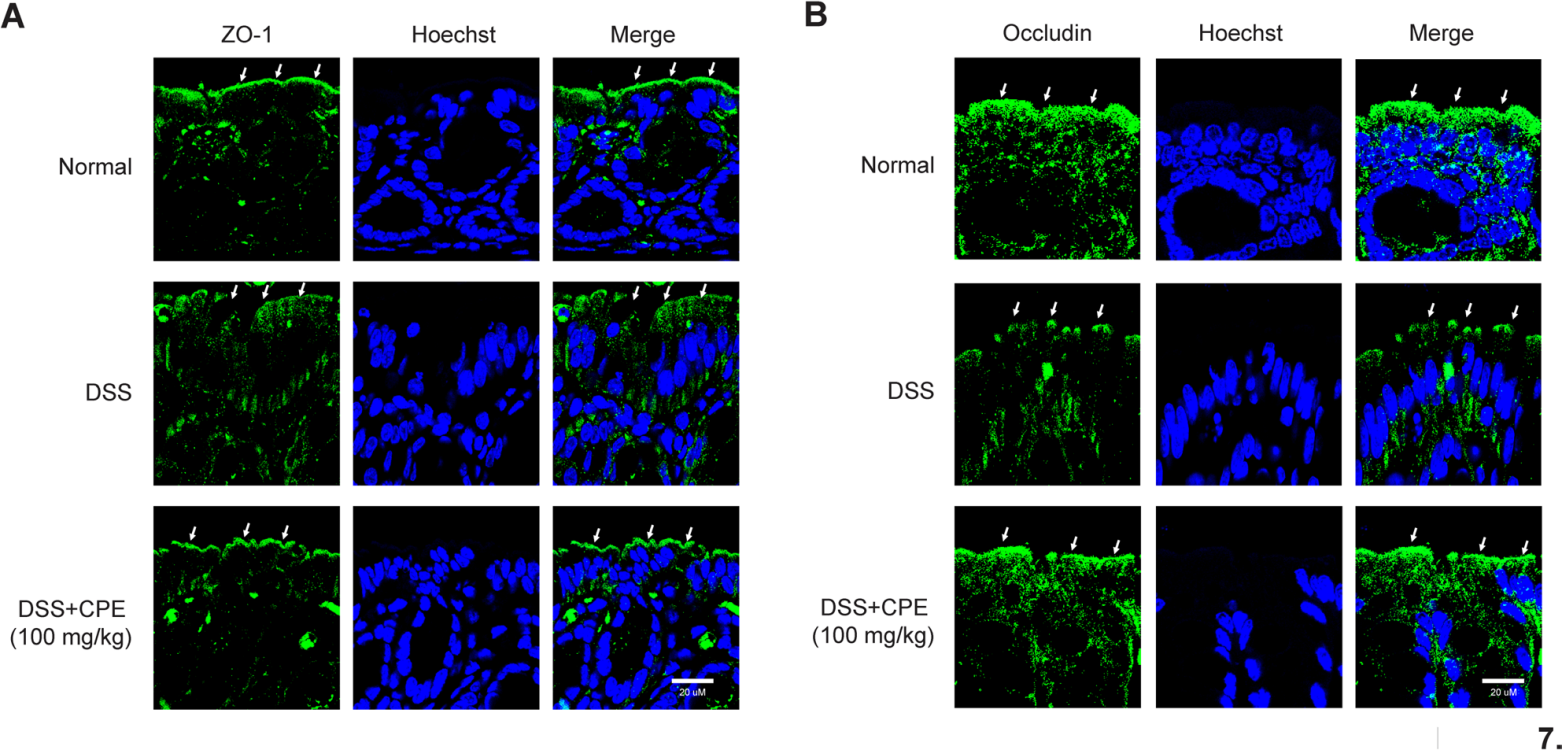
Effects of CPE on tight junction localization of colonic tissues from DSS-induced colitis mice. **A** Immunofluorescence images of ZO-1 localization. ZO-1 localization at the apical junction region was predominantly disrupted in DSS-induced mice compared to normal and CPE treatment fully recovered its localization. **B** Immunofluorescence images of occludin localization. In DSS-fed mice, it was found that occludin was disrupted and was fully re-distributed to apical junction in response to CPE administration

Tight junction proteins form physical barriers at the apical junction region [32]. Therefore, only protein expression profiles of tight junction obtained from western blot analysis may not fully recapitulate barrier property of tight junction in whole tissues. We then further investigated whether CPE induces re-localization of tight junction to apical junction in colitis mice. To achieve this goal, immunofluorescence staining of ZO-1 and occludin were performed. Of note, ZO-1 is considered as a scaffolding protein orchestrating the anastomosed strands of tight junction protein networks and is important for mucosal repair [24, 25, 37, 58]. Conversely, occludin is a transmembrane tight junction protein that maintains intestinal barrier function and is generally disrupted by various cytokines in colitis [6, 28]. Here, it was found that, in colonic tissues of DSS-induced colitis mice, ZO-1 and occludin strands were substantially disrupted when compared to normal mice (Fig. 7A - B). Of particular interest, CPE treatment predominantly promoted re-localization of both ZO-1 and occludin to apical junction of DSS-induced colitis mice (Fig. 7A, B). These data suggested that CPE may be used as a bioactive natural extract that was associated with preservation of tight junction protein expression and localization in this model.

### CPE suppresses tight junction-dependent leak pathway permeability in colitis mouse model

**Fig. 8:**
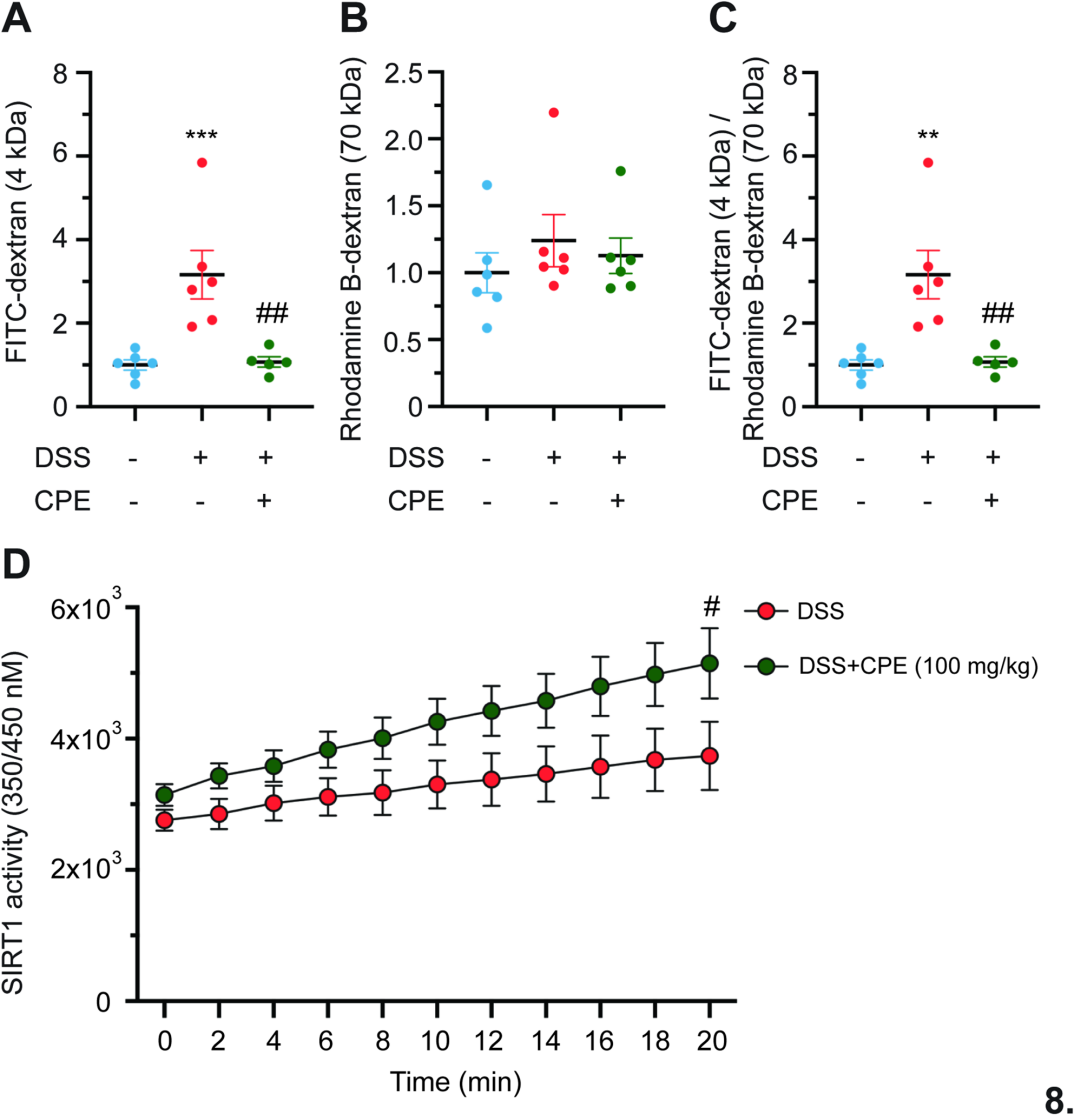
Effects of CPE on tight junction-dependent leak pathway permeability and SIRT-1 activity in colonic tissues of colitis mice. **A** Effect of CPE on permeability rate of 4-kDa FITC-dextran permeability across intestinal barriers in colitis mice. **B** Effect of CPE on permeability rate of 70-kDa Rhodamine B-dextran permeability across intestinal barriers in colitis mice. **C** Permeability ratio of 4-kDa FITC-dextran/70-kDa Rhodamine B-dextran in colitis mice treated with or without CPE treatment. **p < 0.01; ***p < 0.001 compared with normal mice. #*p* < 0.05; ##*p* < 0.01 compared with DSS-treated mice

There are, at least, two types of barrier defects including tight junction-dependent leak pathway permeability and tight junction-independent, tissue damage-related unrestricted pathway permeability that both contribute to colitis pathogenesis and progression [16, 52]. To distinguish the permeability pathway contributing to colitis of our model and to search for the effect of CPE on these permeability pathways, in vivo multiplex permeability assay was performed using a cocktail of FITC-dextran (MW. 4 kDa) and rhodamine-dextran (MW. 70 kDa) as probes for leak pathway permeability and unrestricted pathway permeability [7], respectively. In our DSS-induced colitis mice, the permeability rate of 4-kDa FITC-dextran, but not 70-kDa rhodamine-dextran, was significantly increased when compared to normal mice in consistent with increased ratio of 4-kDa FITC-dextran/70-kDa rhodamine-dextran (Fig. 8A–C). Of particular importance, CPE treatment significantly decreased the permeability rate of 4-kDa FITC-dextran and diminished the ratio of 4-kDa FITC-dextran/70-kDa rhodamine-dextran (Fig. 8A–C). These data strongly suggested that leak pathway permeability was dominant in our mouse model of colitis and CPE effectively suppressed tight junction-dependent leak pathway permeability.

### SIRT-1 activity is upregulated in colitis mice treated with CPE

Our previous transepithelial electrical resistance and western blot results performed in the intestinal epithelial-like T84 cell monolayers indicated that CPE promoted tight junction assembly via SIRT-1-dependent mechanism (Pichayapa [54]). Similar to our in vivo results, we found that CPE treatment in colitis mice significantly enhanced SIRT-1 activity (Fig. 8D). This data implicated the association between SIRT-1 activity and anti-colitogenic effect of CPE.

## Discussion

Here, we reported that CPE attenuated severity of colitis in DSS-induced experimentally acute colitis mouse model. Indeed, we also found that CPE was associated with reduced MLCK transcript abundance, protein expression, and apical localization and NF-κB-mediated inflammatory signaling. In addition, CPE was shown to promote intestinal barrier function, at least in part, by enhancing tight junction re-organization to apical junction and a mechanism associated with SIRT-1 activation. This study may support further preclinical investigation of CPE in experimental intestinal inflammation. However, safety profile, dosing equivalence, and bioavailability of the constituents of CPE need to be further carefully evaluated.

All experiments were performed in male C57BL/6 mice in an acute DSS model. Therefore, this study did not evaluate sex-specific effects and was conducted using an acute murine model, which may not fully recapitulate the initial immune-associated pathophysiology of chronic inflammatory bowel disease in humans. In addition, the absence of safety, pharmacokinetic, bioavailability, and human translational data limits the direct extrapolation of these findings and warrants further investigation in future studies. As depicted in Figs. 1 and 2, CPE was able to relieve all IBD-related symptoms at the physical and histopathological levels. Of particular importance, CPE treatment was capable of reducing mortality in DSS-induced colitis mice (Fig. 1E). The mechanistic studies were focused on the highest effective dose of CPE to ensure interpretability and robustness of the molecular analyses. In this study, we did not, however, identify the effective compounds in CPE that exerts its anti-colitogenic effect. As mentioned above in the introduction part, caffeine, CGA, catechin, epicatechin, and anthocyanins have been demonstrated as major constituents of CPE by previous studies [4, 12, 39, 65]. Similar to several lines of evidence, tentative peak assignments defined by high-performance liquid chromatography (HPLC) analysis suggested that the batch of CPE used in this study mainly contained caffeine, catechin, epicatechin, and CGA (Supplementary Fig. 1, Table 1), but our nuclear magnetic resonance (NMR) spectroscopy data found only caffeine and CGA (Supplementary Fig. 2). These conflicting results might be due to limit of detection (lowest detectable amount), limit of quantitation/quantification (lowest measurable amount with precision), sensitivity differences, and batch variability. These data are consistent with previous evidence regarding anti-colitogenic effects of CGA in DSS-fed mice and high-fat diet rat [17, 67]. Of note, CGA treatment reduced the abundance of *Blautia*, *Sutterella*, and *Akkermansia*, which were inversely correlated to increasing the abundance of butyric acid-producing *Ruminococcus* [67]. Recently, short-chain fatty acid-butyrate supplementation was shown to effectively decrease inflammation in IBD patients and was also able to rescue tissue damage in experimental necrotizing enterocolitis in vitro [14, 15]. In consonance with our previous data, CGA (up to 100 µM) did not directly increase intestinal tight junction assembly in T84 cell monolayers (Pichayapa [54]). Therefore, the possible effect of CPE on gut microbiota changes should be further explored in the next work. Furthermore, epicatechin was reported to protect against bile acid-induced paracellular permeability in Caco-2 monolayers [64]. Nevertheless, direct impacts of catechin and caffeine on intestinal tight junction-dependent barrier function and colitis have never been fully elucidated yet. In fact, coffee and caffeine intake were reported to reduce a risk of ulcerative colitis in Japan [55]. Hence, the pharmacological effects of the major chemicals found in CPE on colitis and inflammation-induced intestinal tight junction disruption need to be addressed.

**Table 1:** Amounts of major compounds found in CPE batch in this study

Compounds	Retention time (min)	Amount (%w/w)
Chlorogenic acid (CGA)	13.406	0.384 ± 0.031
Caffeine	13.944	0.550 ± 0.007
Catechin	9.785	Not detected
Epicatechin	21.208	Not detected

Based on histopathological analyses (Fig. 2), immunofluorescence staining of NF-κB (Fig. 3), and cytokine transcription levels (Fig. 3), we were able to conclude that CPE suppressed colonic inflammation in DSS-induced colitis mice. These results are consistent with several lines of evidence supporting that CPE produced anti-inflammatory effects. Indeed, CPE was shown to protect against polycyclic aromatic hydrocarbons-induced accumulation of intracellular reactive oxygen species (ROS), and suppressed expression of inducible nitric oxide synthase (iNOS), nitric oxide (NO), TNF, IL-6, and cyclooxygenase-2 (COX-2) in macrophages RAW 264.7 cells by inhibiting the p38, MAPK, and NF-κB pathways [40, 44]. There has been currently no report regarding the effect of CPE on MLCK signaling. In this study, we furnished the first time of experimental evidence indicating that CPE significantly decreased transcript and protein expression levels of MLCK. In addition, our immunofluorescence staining revealed that CPE also disrupted inflammation-mediated MLCK recruitment to apical junction of colonic tissues obtained from DSS-induced colitis mice. Although no evidence supporting the role of caffeine, catechin, and epicatechin on MLCK function, CGA was reported to inhibit MLCK/Rho-associated kinase 1 (ROCK1) signaling, resulting in abolishment of LPS- and palmitic acid-induced intestinal barrier disruption in Caco-2 monolayers [51]. Despite no effect on inflammation-mediated decreases in gene expression of *Tjp2* and *Ocln*, our results indicated that CPE recovered protein expression of ZO-1, occludin, and claudin-4 in DSS-induced colitis. Moreover, CPE treatment also promoted re-distribution of ZO-1 and occludin to apical junction after being disrupted in DSS-induced colitis. Furthermore, CPE treatment was demonstrated to specifically suppressed tight junction-dependent leak pathway permeability in DSS-induced colitis as well.

Concerning the possible mechanism of action of CPE, we found that CPE stimulated SIRT-1 activity in colonic tissues of DSS-induced colitis compared to vehicle-treated colitis mice. Currently, there is no evidence supporting the direct inhibitory effect on MLCK function by SIRT-1 activator. However, it is well accepted that activation of NF-κB can upregulate MLCK signaling in various cell types [56, 63, 70]. In consistent with the data in this study, we found that CPE suppressed NF-κB nuclear translocation in colonic tissues. This finding was also supported by previous studies. In fact, antiarthritic drug diacerein and resveratrol were known to inhibit NF-κB activity via SIRT-1-dependent mechanism in diclofenac (DCF)-induced acute nephrotoxicity in rats and *Schistosoma mansoni* (*S. mansoni*)-induced hepatic fibrosis mice [27, 33]. Therefore, SIRT-1 activation by CPE treatment may diminish MLCK signaling by disrupting NF-κB nuclear translocation. Since there are many intracellular signaling molecules that can enhance intestinal tight junction assembly and have long been considered as the anti-colitogenic targets including AMP-activated protein kinase (AMPK), mammalian target of rapamycin (mTOR), and extracellular signal-regulated kinase (ERK) [26, 31, 34, 43], our findings in intestinal epithelial-like T84 monolayers indicated that no involvement of these signaling molecules contributing to CPE-induced intestinal tight junction assembly (Pichayapa [54]). In contrast, CPE-induced occludin and ZO-1 re-distribution to paracellular space in T84 cell monolayers was entirely abolished by pretreatment of SIRT-1 inhibitor (Pichayapa [54]), which was consistent with our data in mouse model of colitis.

## Conclusion

We uncover the pharmacological effects and possible mechanism of action of CPE on DSS-induced colitis mouse models and provides a proof-of-principle that CPE can attenuate severity of colitis. Our findings support the potential of CPE as a cost-effective candidate for further preclinical development in IBD.

## Supplementary Information

Below is the link to the electronic supplementary material.

Supplementary Material 1

Supplementary Material 2

## Data Availability

Data will be made available upon reasonable request.
