# Supplementary material for: Coffea arabica pulp aqueous extract exhibits the anti-colitogenic effect in mice: preventive efficacy and possible mechanisms of action
Source: Biol Res. 2026 May 20;59:41. doi: 10.1186/s40659-026-00696-9 (PMC13366971; doi:10.1186/s40659-026-00696-9)
Supplement: Supplementary file 1 — Supplementary Material 1 [file 40659_2026_696_MOESM1_ESM.pdf]

Supplementary Table 1. Primer sets for quantitative real-time PCR for measurements of cytokine and tight junction transcription.

No.	Transcripts	Primer Sequences (5′ → 3′)	Length	Tm	%GC
1.	*Tjp1*	TGGAATTGCAATCTCTGGTG (Forward)	20	56.01	45
		CTGGCCCTCCTTTTAACACA (Reverse)	20	57.71	50
2.	*Tjp2*	ATGGGAGCAGTACACCGTGA (Forward)	20	60.9	55
		TGACCACCCTGTCATTTTCTTG (Reverse)	22	58.78	45.45
3.	*Ocln*	GCTGTGATGTGTGTGAGCTG (Forward)	20	59.48	55
		GACGGTCTACCTGGAGGAAC (Reverse)	20	59.18	60
4.	*Cldn3*	AAGCCGAATGGACAAAGAA (Forward)	19	54.99	42.11
		CTGGCAAGTAGCTGCAGTG (Reverse)	19	58.83	57.89
5.	*Cldn4*	CGCTACTCTTGCCATTACG (Forward)	19	56.24	52.63
		ACTCAGCACACCATGACTTG (Reverse)	20	58.11	50
6.	*Cldn7*	AGGGTCTGCTCTGGTCCTT (Forward)	19	59.84	57.89
		GTACGCAGCTTTGCTTTCA (Reverse)	19	56.86	47.37
7.	*Cldn8*	GCCGGAATCATCTTCTTCAT (Forward)	20	55.59	45
		CATCCACCAGTGGGTTGTAG (Reverse)	20	57.89	55
8.	*Il1b*	GAGTGTGGATCCCAAGCAAT (Forward)	20	57.87	50
		TACCAGTTGGGGAACTCTGC (Reverse)	20	59.31	55
9.	*Il6*	TAGTCCTTCCTACCCCAATTTCC (Forward)	23	59.22	47.83
		TTGGTCCTTAGCCACTCCTTC (Reverse)	21	59.37	52.38
10.	*Tnf*	AGCCCCCAGTCTGTATCCTT (Forward)	20	59.96	55
		GGTCACTGTCCCAGCATCTT (Reverse)	20	59.67	55
11.	*MYLK*	TCTCCTGCAAGATCACTGGC (Forward)	20	62.2	55
		CTCAGACACAGACACACGGG (Reverse)	20	63.6	60
12.	*Il8*	CGCCCAGACAGAAGTCATAG (Forward)	20	57.78	55
		TCCTCCTTTCCAGGTCAGTTA (Reverse)	21	57.73	47.62
13.	*GAPDH*	GCCAAGAGGGTCATCATCTC (Forward)	20	57.75	55
		CCTTCCACAATGCCAAAGTT (Reverse)	20	56.79	45

Supplementary Table 2. Lists of antibodies used for western blot analysis and immunofluorescence staining

No.	List of antibodies	Vendor	Cat. #
1.	Rabbit anti-NF-κB p65 monoclonal antibody (Clone: D14E12)	Cell Signaling	8242S
2.	Mouse anti-MLCK monoclonal antibody (Clone: K36)	Sigma-Aldrich	M7905
3.	Rabbit anti-Claudin-1 monoclonal antibody (Clone: D3H7C)	Cell Signaling	13995S
4.	Mouse anti-Claudin-4 monoclonal antibody (Clone: 3E2C1)	ThermoFisher scientific	32-9400
5.	Mouse anti-ZO-1 monoclonal antibody (Clone: ZO1-1A12)	ThermoFisher scientific	33-9100
6.	Mouse anti-Occludin monoclonal antibody (Clone: OC-3F10)	ThermoFisher scientific	33-1500
7.	Rabbit anti-β-Actin monoclonal antibody (Clone: 13E5)	Cell Signaling	4970S
8.	Anti-rabbit IgG, HRP-linked antibody	Cell Signaling	7074S
9.	Anti-mouse IgG, HRP-linked antibody	Cell Signaling	7076S
10.	Goat Anti-Mouse IgG H&L (Alexa Fluor® 488)	Abcam	Ab150113
11.	Goat Anti-Rabbit IgG H&L (Alexa Fluor® 594)	Abcam	Ab150080

A

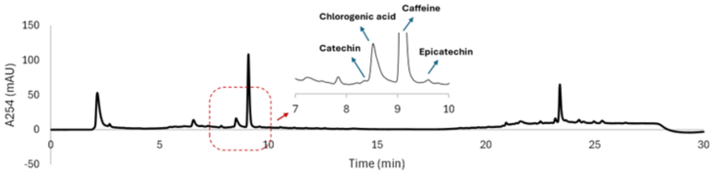

Compound	RT (min)		
	Standard	Found in the crude extract	Relative % area
Caffeine	9.051	9.088	71.4
Catechin	8.349	8.352	2.5
Epicatechin	9.533	9.603	4.8
chlorogenic acid	8.505	8.523	21.3

B

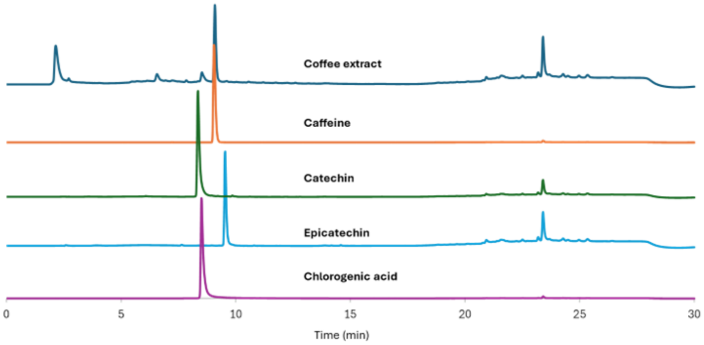

Supplementary Figure 1. The HPLC analysis of the *coffee arabica* pulp aqueous extract. A) The chromatogram showing peaks at 254 nm of various compounds in the extract. B) Stacking chromatograms from the extract and each standard compounds, caffeine, catechin, epicatechin, and chlorogenic acid.

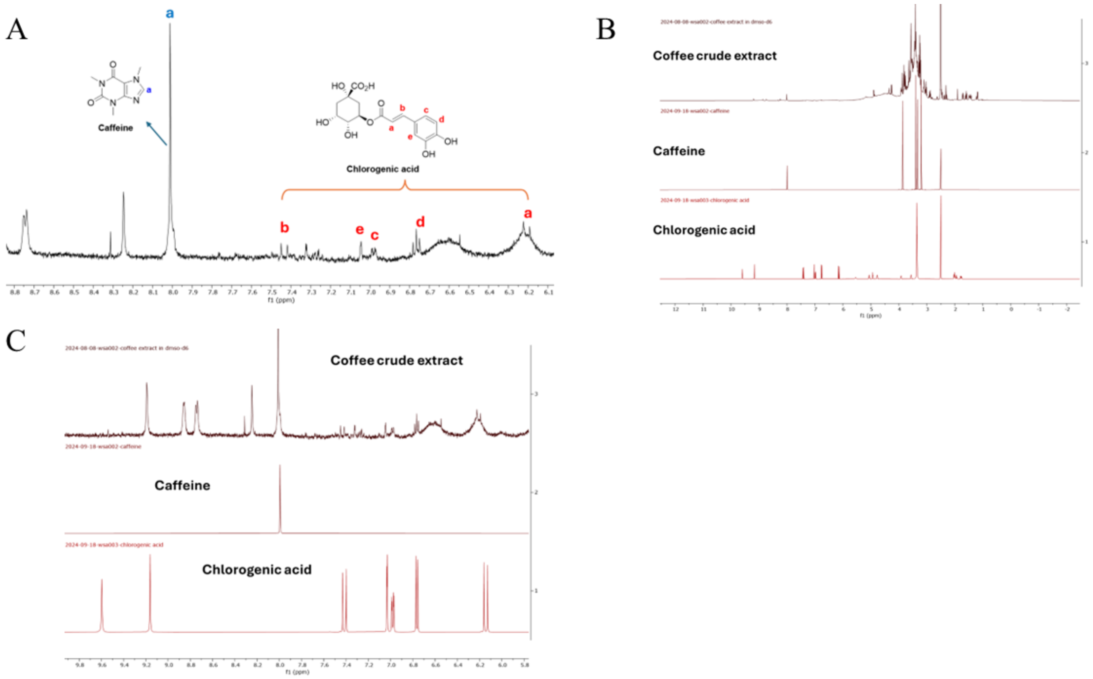

Supplementary Figure 2. The H1 NMR analysis of the *coffee arabica* pulp aqueous extract. A) The NMR spectrum showing various peaks that belong to caffeine and chlorogenic acid as labelled a (in blue for the caffeine) and a – e (in red for the chlorogenic acid) in the extract. B) Stacking NMR spectra for the extract and each standard compounds, caffeine and chlorogenic acid. C) Zoom-in version of the stacking NMR spectra from B) between 5.8 – 9.8 ppm.
