# Supplementary material for: Coffea arabica pulp aqueous extract exhibits the anti-colitogenic effect in mice: preventive efficacy and possible mechanisms of action
Source: Biol Res. 2026 May 20;59:41. doi: 10.1186/s40659-026-00696-9 (PMC13366971; doi:10.1186/s40659-026-00696-9)

Western blot bands of CPE reduced **MLCK** protein expression in DSS-induced colitis mice.

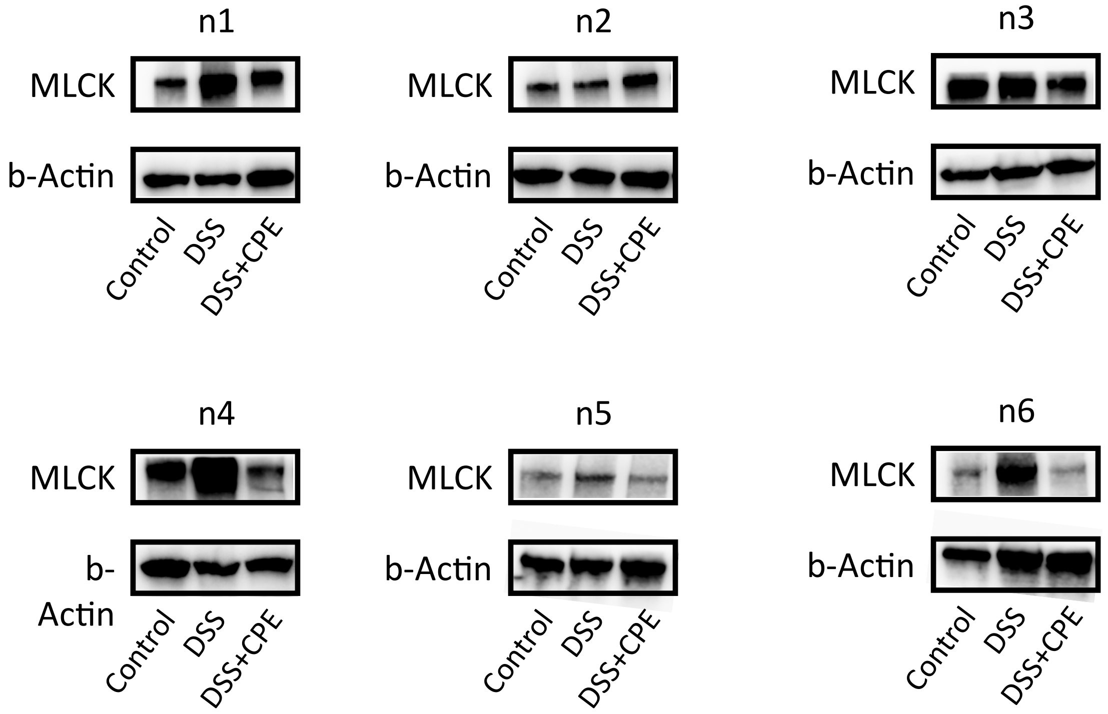

Western blot bands of CPE reduced **ZO-1** protein expression in DSS-induced colitis mice.

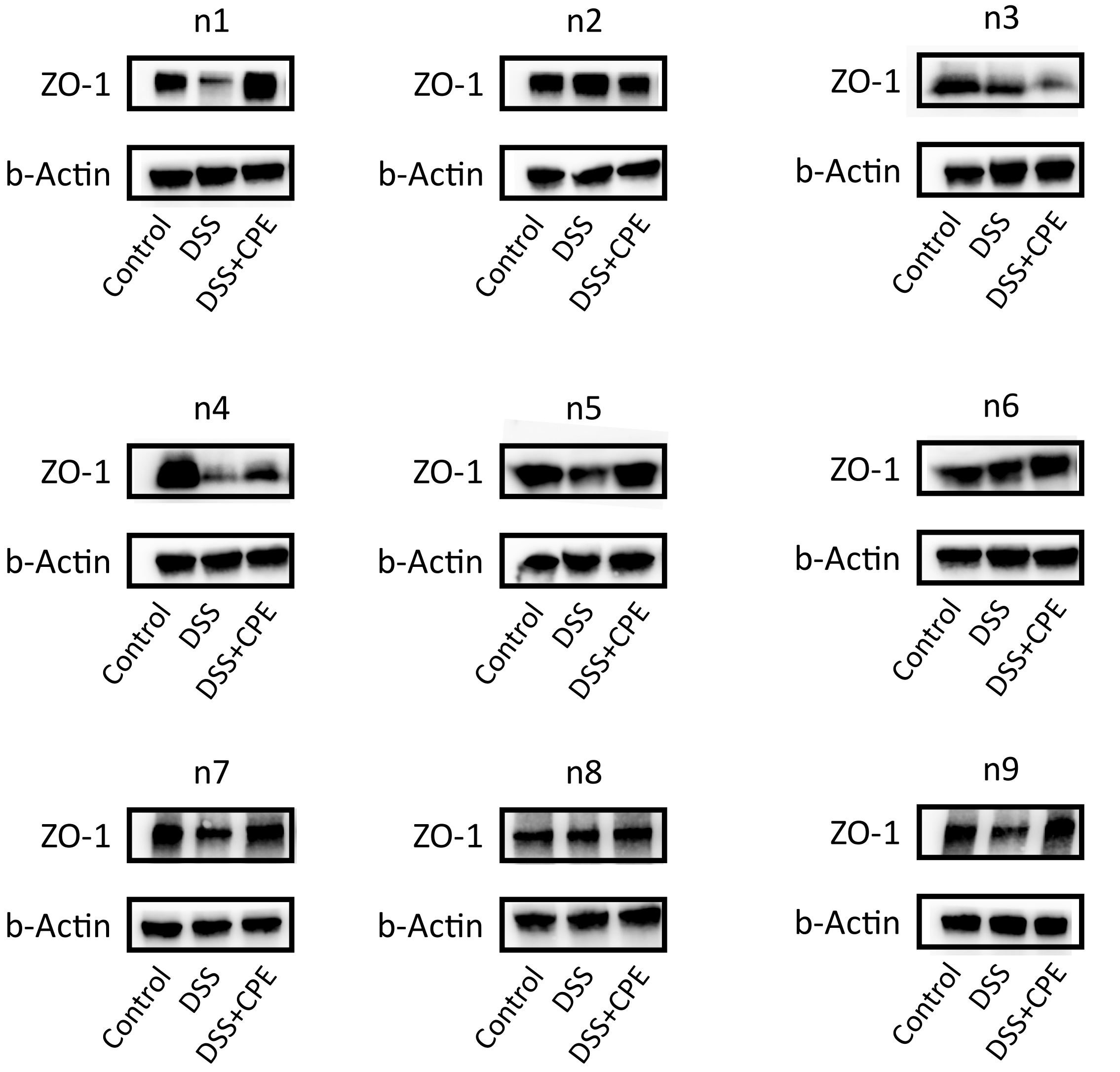

Western blot bands of CPE reduced **Occludin** protein expression in DSS-induced colitis mice.

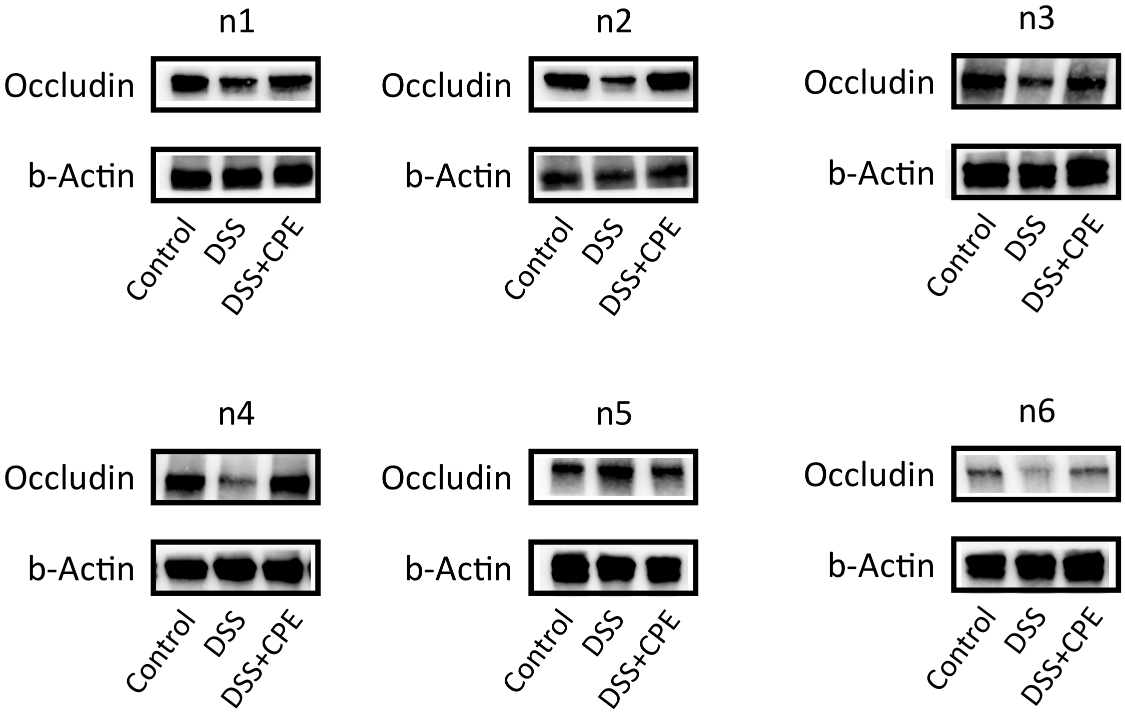

Western blot bands of CPE reduced **Claudin-1** protein expression in DSS-induced colitis mice.

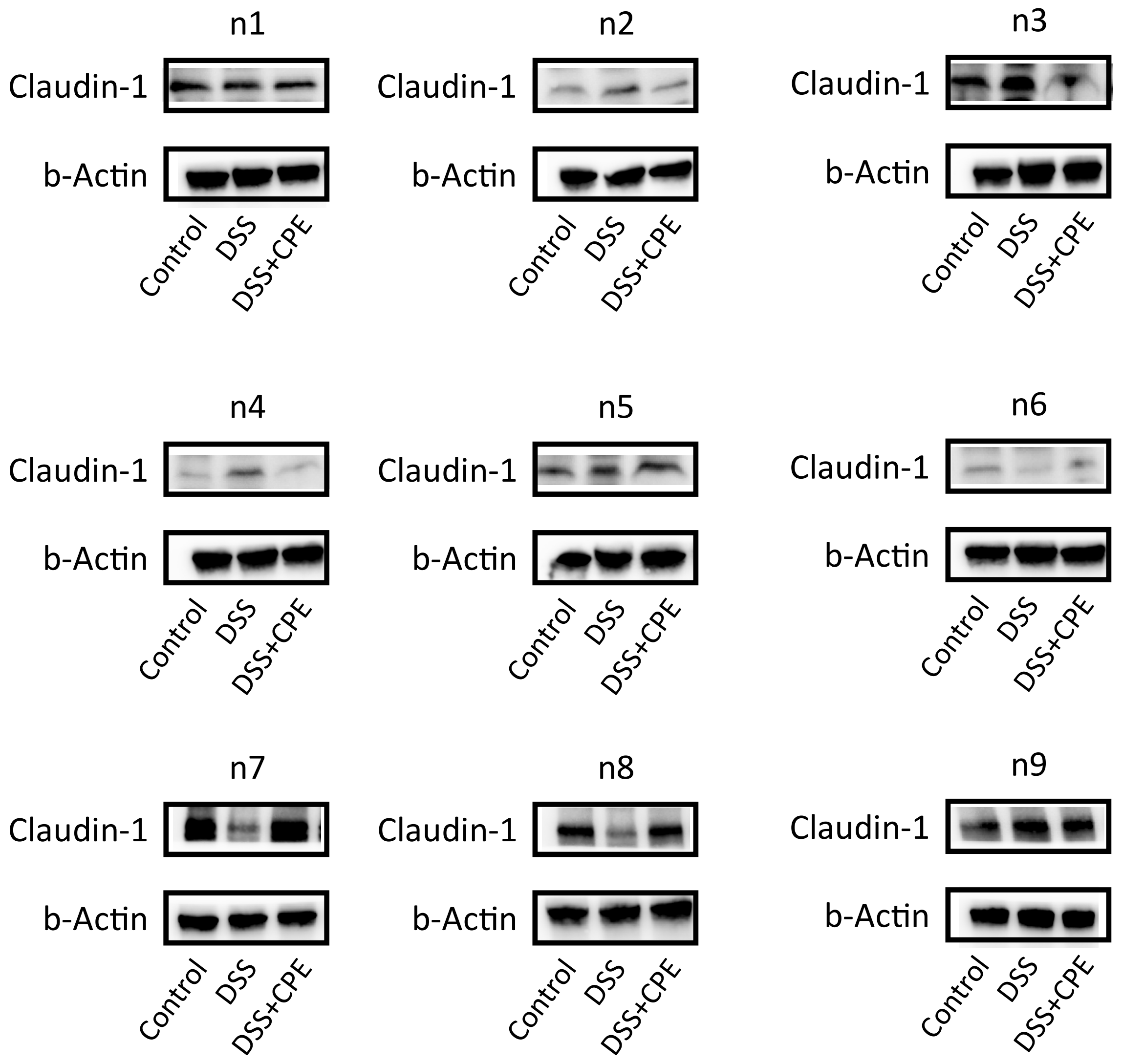

Western blot bands of CPE reduced **Claudin-4** protein expression in DSS-induced colitis mice.

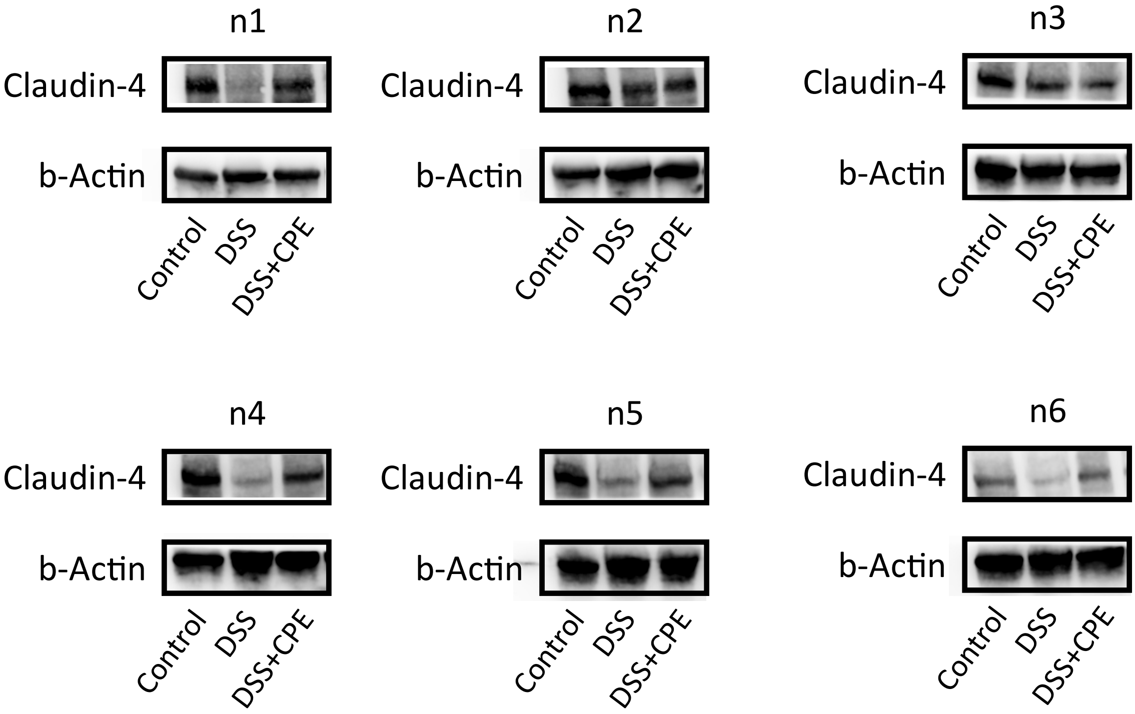

Supplement: Supplementary file 2 — Supplementary Material 2 [file 40659_2026_696_MOESM2_ESM.pdf]
